# *Sirt1* AS lncRNA interacts with its mRNA to inhibit muscle formation by attenuating function of miR-34a

**DOI:** 10.1038/srep21865

**Published:** 2016-02-23

**Authors:** Guo-qiang Wang, Yu Wang, Yan Xiong, Xiao-Chang Chen, Mei-ling Ma, Rui Cai, Yun Gao, Yun-mei Sun, Gong-She Yang, Wei-Jun Pang

**Affiliations:** 1 Laboratory of Animal Fat Deposition & Muscle Development, College of Animal Science and Technology, Northwest A&F University, Yangling Shaanxi 712100, China

## Abstract

Recent studies demonstrate the functions of long non-coding RNAs (lncRNAs) in mediating gene expression at the transcriptional or translational level. Our previous study identified a *Sirt1* antisense (AS) lncRNA transcribed from the *Sirt1* AS strand. However, its role and regulatory mechanism is still unknown in myogenesis. Here, functional analyses showed that *Sirt1* AS lncRNA overexpression promoted myoblast proliferation, but inhibited differentiation. Mechanistically, *Sirt1* AS lncRNA was found to activate its sense gene, *Sirt1*. The luciferase assay provided evidences that *Sirt1* AS lncRNA interacted with *Sirt1* 3′ UTR and rescued *Sirt1* transcriptional suppression by competing with miR-34a. In addition, RNA stability assay showed that *Sirt1* AS lncRNA prolonged *Sirt1* mRNA half-life from 2 to 10 h. Ribonuclease protection assay further indicated that it fully bound to *Sirt1* mRNA in the myoblast cytoplasm. Moreover, *Sirt1* AS overexpression led to less mouse weight than the control because of less lean mass and greater levels of *Sirt1*, whereas the fat mass and levels of miR-34a were not altered. Based on the findings, a novel regulatory mechanism was found that *Sirt1* AS lncRNA preferably interacted with *Sirt1* mRNA forming RNA duplex to promote *Sirt1* translation by competing with miR-34a, inhibiting muscle formation.

Recently, long noncoding RNA (lncRNA) has been shown to play important functional roles as regulators of gene expression through the recruitment of the complex epigenetic machinery that dictates distinctive chromatin signatures involved in active transcription and translation. One group of lncRNAs is the natural antisense transcripts (NATs), which are transcribed from the opposite DNA strand to their specific partner protein-coding genes. Antisense transcription is the pairing of a NAT with an overlapping protein-coding sense transcript, whereby NAT expression can lead to an increase or decrease in sense expression^1^.

Muscle development is orchestrated by a network of epigenetic regulators and transcription factors^2^,^3^,^4^,^5^. LncRNA emerges as an important novel player in muscle development regulation^6^. LncRNA regulates key function genes by numbers of mechanisms at different levels, including transcriptional, post-transcriptional and epigenetic^7^. At the transcriptional level, muscle-associated lincRNA (Yam-1) was decreased during muscle differentiation and served as a repressor of myogenesis^8^. In addition, endogenous RNA (eRNA) was another class of ncRNA which was transcribed from regulatory elements^9^. The eRNA transcribed from the regulatory elements of myogenic differentiation antigen (*MyoD*), which enhanced RNA polymerase II occupancy and transcription of *MyoD* and myogenin (*MyoG*), further activated the downstream myogenic effects^10^. At the post-transcriptional level, the muscle-specific lncRNA (linc-MD1) ‘‘sponges’’ miR-133 and miR-135 to regulate the expression of transcription factors mastermind-like protein 1 (MAML1) and myocyte-specific enhancer factor 2C (MEF2C) to activate muscle-specific gene expression^11^. At the epigenetic level, lncRNAs including metastasis-associated lung adenocarcinoma transcript 1 (Malat1), H19 and gene trap locus 2-maternally expressed gene 3 (*Gtl2*-*Meg3*) interacted with polycomb repressive complex 2 (PRC2) to modulate their target genes, resulting in myogenesis^12^,^13^,^14^. However, whether other novel lncRNAs are involved in muscle development needs to further explore.

According to sequence length, ncRNAs were classified as lncRNA (>200 bp) and microRNA (~22 bp)^7^. Increasing evidences verified that lncRNA or microRNA interacted with each other to fulfil the biological function. For example, linc-MD1 and H19 sponge miR-133, miR-135 and let7, respectively, inhibiting the function of miRNAs^15^. In addition, some lncRNAs directly regulated the processing of miRNAs. LncRNA (Uc.283 + A) bound to pri-miR-195 to prevent its cleavage and maturation^16^. Moreover, lncRNAs indirectly regulated the processing of miRNAs. MiR-361 targeted pri-miR-484 to suppress the processing of miR-484, but mitochondrial dynamic related lncRNA (MDRL) bound to miR-361 and indirectly promoted miR-484 maturation^17^. Therefore, it is necessary to further explore the novel mechanisms of interaction between lncRNA and miRNA.

More than 70% of mammalian transcripts show evidence of AS transcription not only indicates the biological importance of NATs but could also have various functional implications. Very recently, our group has successfully performed interference of *PU.1* (also known as *SPI1*) AS lncRNA to substantially promote *PU.1* mRNA translation, leading to inhibition of adipogenesis^18^. We have now identified a novel NAT that corresponds to Sirtuin type 1 (*Sirt1*) AS lncRNA.

*Sirt1*, a NAD-dependent class III protein deacetylases, is a member of sirtuins family^19^. Some studies suggested that *Sirt1* regulated the balance between myoblast proliferation and differentiation^20^. Upregulation of *Sirt1* promoted muscle precursor cell proliferation by inhibiting cell cycle inhibitor expression^21^,^22^. On the contrary, *Sirt1* suppressed myoblasts differentiation through deacetylation of MEF2^23^. Therefore, *Sirt1* has opposite effects in myoblast proliferation and differentiation. Precise regulation of *Sirt1* expression is crucial to balance myoblast proliferation and differentiation. Our previous study identified that *Sirt1* AS lncRNA transcribed from *Sirt1* AS strand and overlapped with *Sirt1* mRNA 3′ untranslated region (3′ UTR)^24^, but its function and mechanism in myogenesis are still unclear.

In this study, the proliferation and differentiation were further detected in myoblasts of *Sirt1* AS or/and miR-34a overexpression, and regulatory mechanism were explored using the dual luciferase reporter, RNA stability and ribonuclease protection assay, as well the mice of *Sirt1* AS overexpression were studied by intraperitoneal injection of adenovirus on their weights, body composition and muscle fiber characteristics. Our findings provided a novel regulatory mechanism: it was that *Sirt1* AS lncRNA preferably interacted with *Sirt1* mRNA forming RNA duplex by competing with miR-34a to inhibit muscle formation.

## Results

### The expression patterns of *Sirt1* mRNA, *Sirt1* AS lncRNA and miR-34a during myoblast proliferation

**Figure 1:**
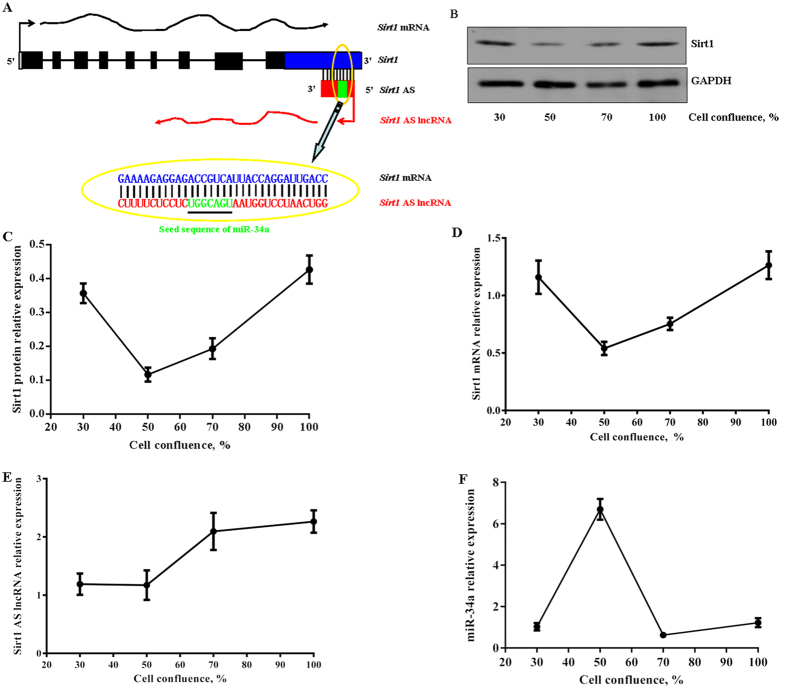
Expression of *Sirt1* AS lncRNA, mRNA and miR-34a during C2C12 myoblast proliferation. (**A**) Putative schematic of mouse miR-34a and bidirectional transcriptional *Sirt1* transcripts including mRNA and AS lncRNA. (**B,C**) Expression of *Sirt1* protein. (**D**) Expression of *Sirt1* mRNA. (**E**) Expression of *Sirt1*AS lncRNA. (**F**) Expression of miR-34a.

Our previous study showed the stability and expression of *Sirt1* AS lncRNA overlapped with partial of *Sirt1* 3′ UTR^24^. Interestingly, in this study, we found that the target sequence of miR-34a to *Sirt1* mRNA was in the overlapping region (Fig. 1A). To examine the expression patterns of *Sirt1* AS lncRNA, *Sirt1* mRNA and miR-34a, their levels were detected by qPCR or western blotting during myoblast proliferation, respectively. The results indicated that the levels of *Sirt1* protein were concordant with *Sirt1* mRNA, which decreased in 50% cell confluence and increased from 70% to 100% cell confluence (Fig. 1B–D). In addition, the levels of *Sirt1* AS lncRNA gradually increased during myoblast proferation (Fig. 1E), whereas the levels of miR-34a increased in 50% cell confluence and decreased in 70% cell confluence (Fig. 1F).

### *Sirt1* AS lncRNA attenuated the inhibition of *Sirt1* expression against miR-34a

**Figure 2:**
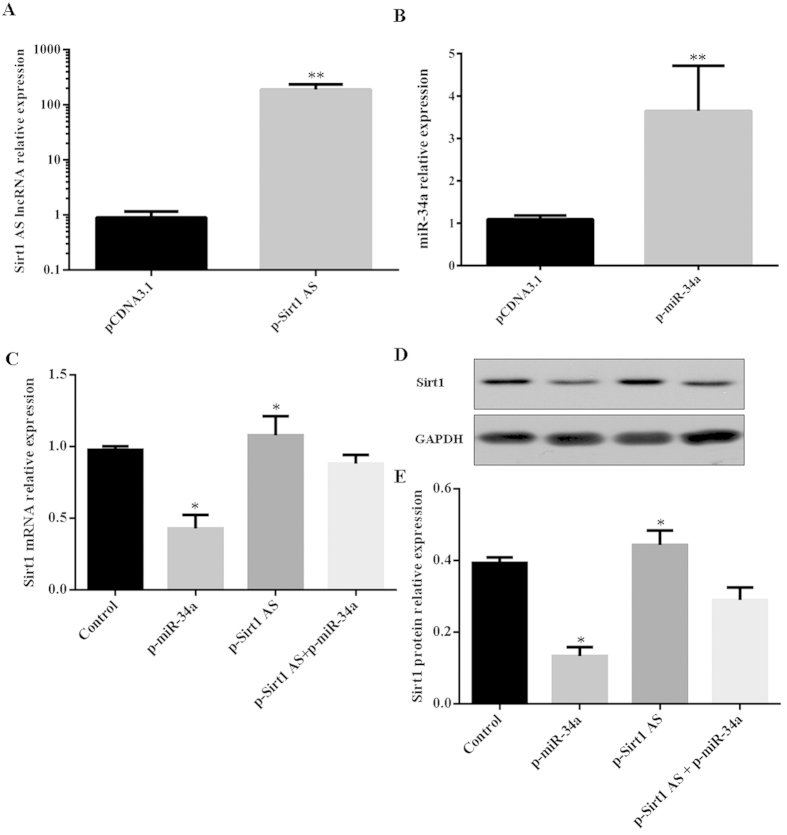
S*irt1* AS overexpression recovered the levels of *Sirt1* mRNA and protein against the function of miR-34a in C2C12 myoblasts. (**A**) *Sirt1* AS overexpression in myoblasts. *GAPDH* was employed as an internal reference. (**B**) miR-34a overexpression. (**C**) The levels of *Sirt1* mRNA in myoblasts transfected with p-*Sirt1* AS (pCDNA3.1-*Sirt1* AS), p-miR-34a (pCMV-miR-34a) and p-*Sirt1* AS plus p-miR-34a. (**D,E**) The levels of *Sirt1* protein in myoblasts transfected with p-*Sirt1* AS, p-miR-34a and p-*Sirt1* AS plus p-miR-34a. The levels of *Sirt1* protein were detected by western blotting. GAPDH protein was applied as internal reference. The data were presented as means ± SEM of 4 independent experiments. *P < 0.05 and **P < 0.01.

To explore the regulation of *Sirt1* AS lncRNA and miR-34a to *Sirt1* expression, *Sirt1* AS and miR-34a expression plasmids were constructed. The results showed that expression efficiencies of *Sirt1* AS and miR-34a were about 200-fold and 4-fold in myoblasts, respectively (Fig. 2A,B). In addition, *Sirt1* AS lncRNA was elevated more than 40-fold by 100 μM resveratrol using strand-specific RT-PCR analysis (Fig. S1). Furthermore, miR-34a overexpression downregulated the levels of *Sirt1* mRNA and protein, whereas *Sirt1* AS overexpression upregulated the levels of *Sirt1* mRNA and protein (Fig. 2C–E). Interestingly, the levels of *Sirt1* mRNA and protein were rescued when *Sirt1* AS and miR-34a were co-overexpressed (Fig. 2C–E).

### *Sirt1* AS lncRNA promoted myoblast proliferation

**Figure 3:**
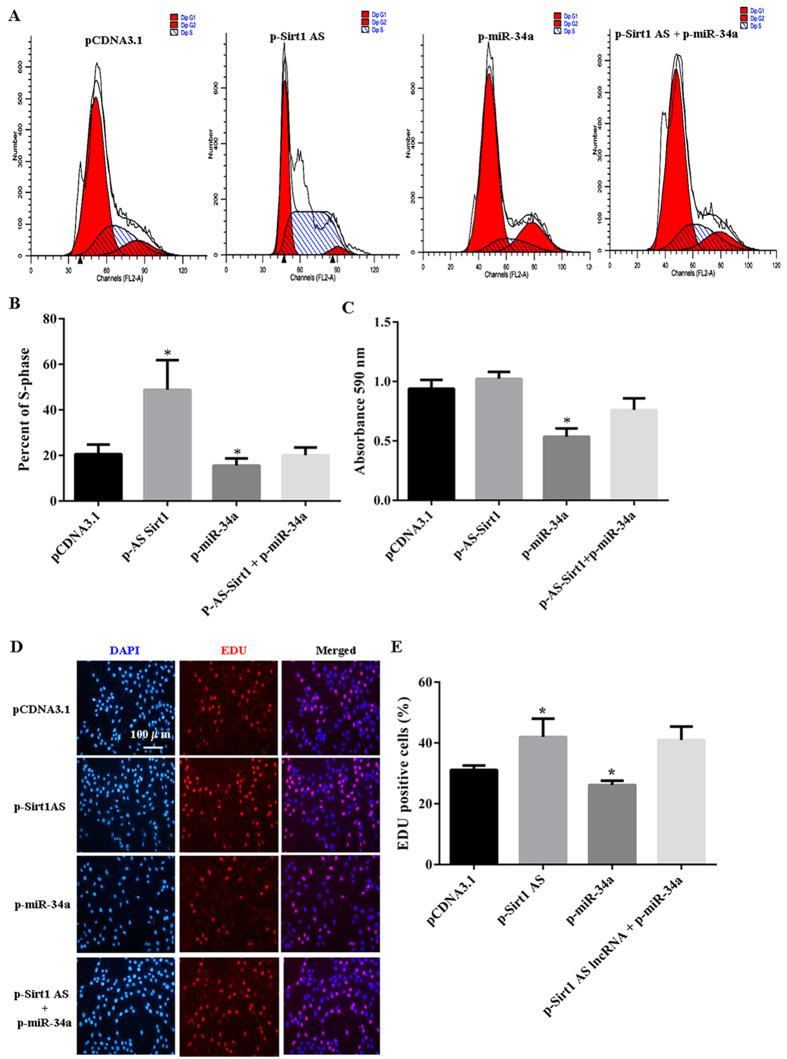
S*irt1* AS overexpression promoted myoblast proliferation. (**A**,**B**) Cell cycle analysis using flow cytometry for myoblasts of *Sirt1* AS, miR-34a and *Sirt1* AS plus miR-34a overexoression. (**C**) CCK-8 cell proliferation analysis. (**D**) EdU labeling and immunocytochemical staining of proliferating cells. (**E**) The quantification analysis of EdU-positive cells. Results were indicated as the mean ± SEM of 4 independent experiments. *P < 0.05.

To discover the functional *Sirt1* AS lncRNA associated with myoblast proliferation, we detected proliferation of the cells transfected with *Sirt1* AS expression vector by flow cytometry, cell counting kit-8 (CCK-8) and 5-ethynyl-2′- deoxyuridine (EDU) analysis. As shown in Fig. 3A,B, *Sirt1* AS overexpression increased the number of cells which were in DNA synthesis (S) phage of cell cycle, whereas miR-34a overexpression decreased this number. Furthermore, the ability of myoblasts to proliferation was rescued when *Sirt1* AS and miR-34a were co-overexpressed (Fig. 3A,B). The CCK8 detection confirmed that *Sirt1* AS lncRNA was against inhibiting myoblast proliferation of miR-34a (Fig. 3C). In addition, the above results were further verified by EDU assay as well. The ratio of EDU positive cells indicated the cell in DNA synthesis phage. *Sirt1* AS overexpression increased the ratio of EDU positive cells and resisted the inhibitory myoblast proliferation of miR-34a (Fig. 3D,E).

### *Sirt1* AS lncRNA rescued the expression of proliferation genes by resisting miR-34a

**Figure 4:**
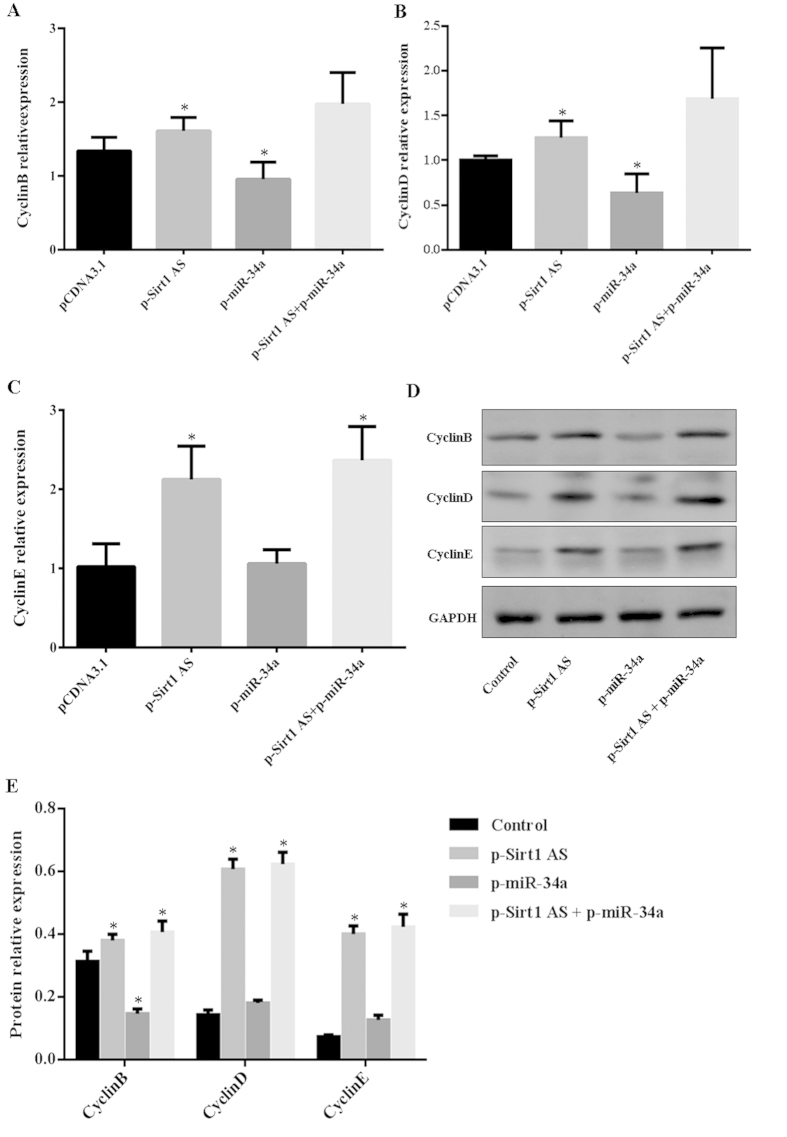
*Sirt1* AS overexpression increased the levels of Cyclins. (**A**–**C**) The mRNA levels of *CyclinB*, *CyclinD* and *CyclinE* were upregulated in C2C12 cells of *Sirt1* AS overexpression, but downregulated in miR-34a overexpression at 24 hours after treatment with growth medium. (**D,E**) The levels of CyclinB, CyclinD and CyclinE proteins in C2C12 cells of *Sirt1* AS, miR-34a and *Sirt1* AS plus miR-34a overexoression at 24 hours after induced with growth medium. Results were indicated as the mean ± SEM of 4 independent experiments. *P < 0.05.

To further elucidate the regulation at gene level, the expression of proliferation genes were examined in C2C12 cells transfected with the expression vectors of *Sirt1* AS, miR-34a and both of them, respectively. The results indicated that *Sirt1* AS overexpression upregulated the mRNA levels of *CyclinB*, *CyclinD* and *CyclinE*, but miR-34a overexpression downregulated the mRNA levels of *CyclinB* and *CyclinD* (Fig. 4A–C). Moreover, *Sirt1* AS overexpression rescued the levels of *CyclinB*, *CyclinD* and *CyclinE* when *Sirt1* AS and miR-34a were co-overexpressed (Fig. 4A–C). In addition, western blotting analysis of CyclinB, CyclinD and CyclinE further confirmed that *Sirt1* AS lncRNA promoted their expression and attenuated the inhibition of miR-34a on the expression of proliferation marker genes (Fig. 4D,E).

### *Sirt1* AS lncRNA inhibited myoblast differentiation

**Figure 5:**
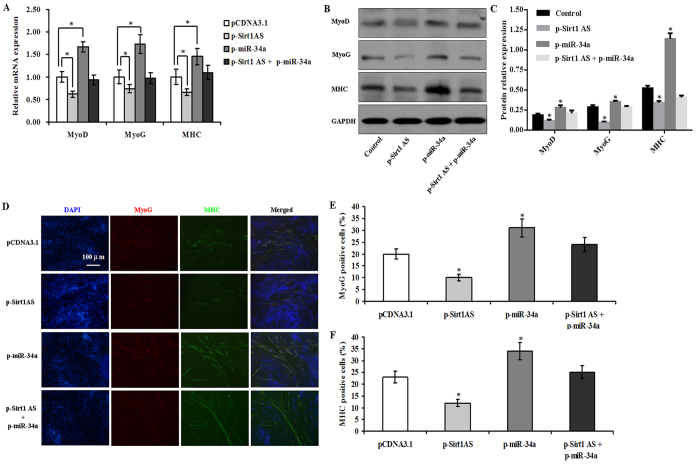
*Sirt1* AS overexpression inhibited myoblast differentiation. (**A**–**C**) *Sirt1* AS overexpression downregulated the levels of myogenic gene (*MyoD*, *MyoG* and *MHC*) mRNA and protein. (**D**) Immunocytochemical staining. (**E,F**) The quantification analysis of MyoG and MHC positive nuclei. The data were presented as means ± SEM of 4 independent experiments. *P < 0.05.

Upregulation of *Sirt1* protein level by *Sirt1* AS lncRNA suggested that it may be an inhibitory myogenic factor during myoblast differentiation. To investigate *Sirt1* AS lncRNA relevancy in myogenesis, we overexpressed *Sirt1* AS in C2C12 cells. The results showed that *Sirt1* AS overexpression downregulated the levels of *MyoD*, *MyoG* and myosin heavy chain (*MHC*) mRNA and protein, whereas miR-34a overexpression upregulated the levels of above gene expression (Fig. 5A–C). Interestingly, *Sirt1* AS overexpression decreased the levels of these genes when both *Sirt1* AS and miR-34a were co-overexpressed (Fig. 5A–C). Moreover, the results were also strengthened by immunofluorescence staining for MHC and MyoG protein in differentiating myotubes on day 2 post-transfection (Fig. 5D). In addition, the number of positive myotubes was reduced by *Sirt1* AS overexpression, but was increased by miR-34a overexpression (Fig. 5E,F). Interestingly, the number of positive myotubes was recovered by co-overexpression of both *Sirt1* AS and miR-34a (Fig. 5E,F).

### *Sirt1* AS lncRNA increased the stability of *Sirt1* mRNA by binding to its 3′ UTR via competing with miR-34a

**Figure 6:**
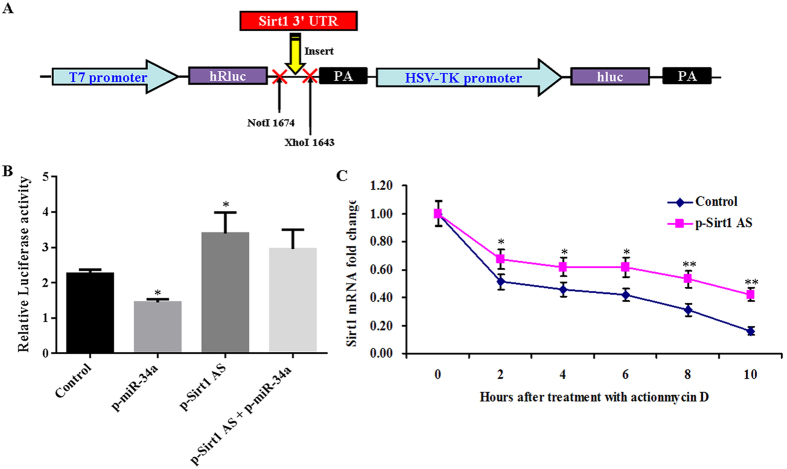
*Sirt1* AS lncRNA promoted transcription of *Sirt1* by attenuating the role of miR-34a. (**A**) Schematic of *Sirt1* 3′ UTR inserted into psiCHECK™-2 vector. (**B**) Luciferase activity analysis for *Sirt1* AS, *Sirt1* and miR-34a. (**C**) *Sirt1* AS lncRNA makes *Sirt1* mRNA much more stable. The data were presented as means ± SEM of 4 independent experiments. *P < 0.05 and **P < 0.01.

To probe into the mechanism underlying the myogenic-mediated function of *Sirt1* AS lncRNA, we constructed *Sirt1* 3′ UTR psiCHECK™-2 vector for luciferase reporter assay (Fig. 6A). The results indicated that *Sirt1* AS lncRNA increased relative luciferase activity by directly interacting with *Sirt1* 3′ UTR in 293T cells transfected with *Sirt1* AS expression vector, whereas miR-34a decreased relative luciferase activity by targeting *Sirt1* 3′ UTR in 293T cells transfected with miR-34a expression vector (Fig. 6B). Compare with miR-34a transfection, *Sirt1* AS lncRNA partially rescued the relative luciferase activity in 293T cells co-transfected with *Sirt1* AS lncRNA and miR-34a (Fig. 6B). Therefore, our results suggested that *Sirt1* AS lncRNA bound to *Sirt1* 3′ UTR by competing with miR-34a to regulate *Sirt1* gene transcription. Moreover, the RNA stability assay illustrated that *Sirt1* AS overexpression prolonged *Sirt1* mRNA half-life from 2 to 10 h after treatment with actionmy D (Fig. 6C), implying that *Sirt1* AS lncRNA increased the stability of *Sirt1* mRNA though interacting with *Sirt1* 3′ UTR by competing with miR-34a.

**Figure 7:**
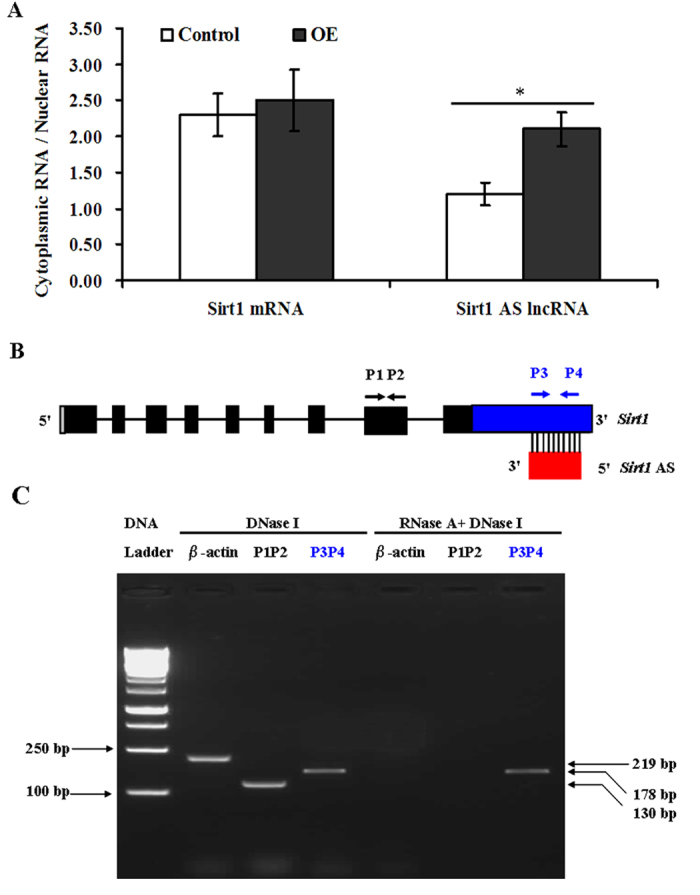
*Sirt1* AS lncRNA fully bound to *Sirt1* mRNA on basement of basyl complementary pairing principle in myoblasts. (**A**) *Sirt1* AS lncRNA and *Sirt1* mRNA existed in cytoplasm and nucleus. *P < 0.05. (**B**) Primer positions for ribonuclease protection assay (RPA). (**C**) Detection of the *Sirt1* AS lncRNA/mRNA pairs. OE: *Sirt1* AS overexpression.

To directly confirm the interaction between *Sirt1* AS lncRNA and *Sirt1* mRNA, ribonuclease protection assay (RPA) and RT-PCR were performed on the cytoplasmic RNA from C2C12 cells. We found that the ratio of cytoplasmic *Sirt1* mRNA/nuclear *Sirt1* mRNA did not changed after *Sirt1* AS overexpression, but the ratio of cytoplasmic *Sirt1* AS lncRNA/nuclear *Sirt1* AS lncRNA increased (Fig. 7A). Furthermore, the RT-PCR primers were designed according to putative schematic of *Sirt1* AS lncRNA and *Sirt1* mRNA (Fig. 7B). RPA showed that cytoplasmic sense/antisense RNA duplex formation occured was fully overlapping between the two strands, resulting in protection (Fig. 7C).

### *Sirt1* AS lncRNA inhibited muscle formation

**Figure 8:**
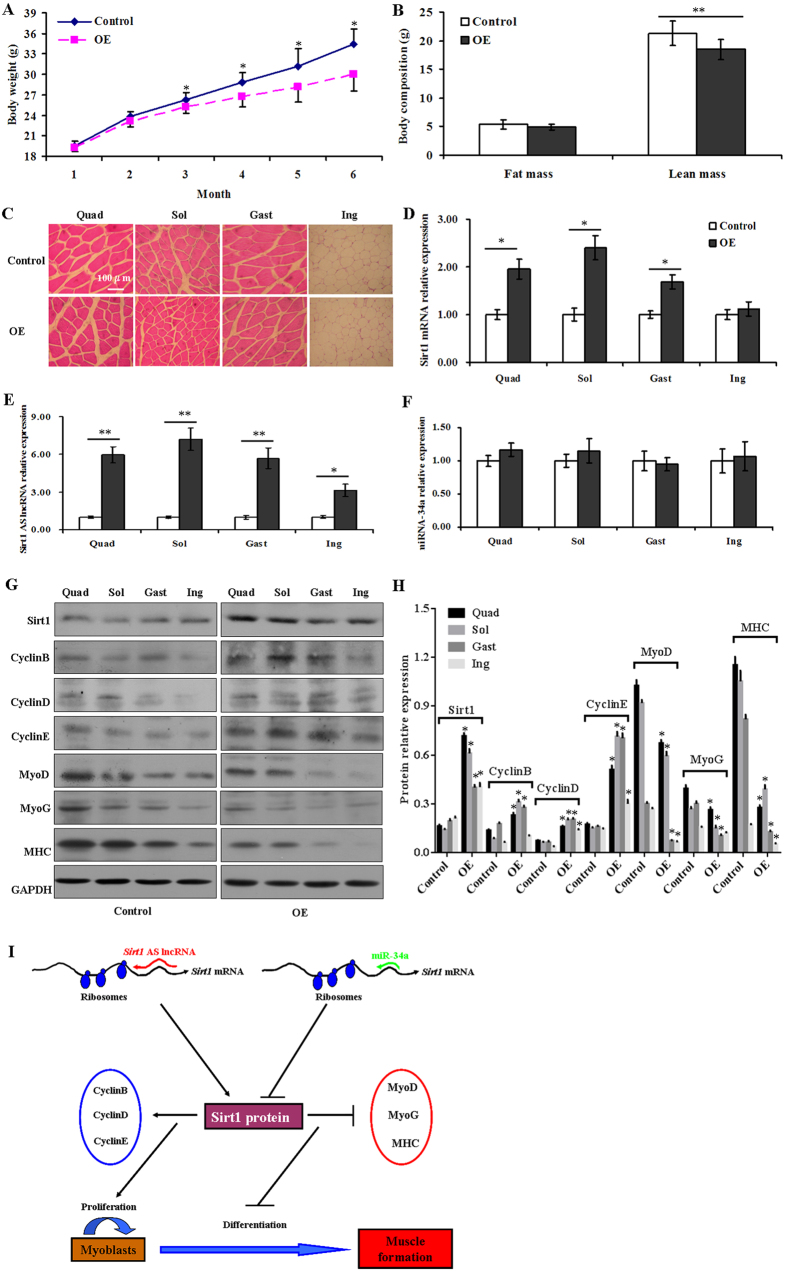
*Sirt1* AS overexpression inhibited muscle development. (**A**) Body weight. (**B**) Body composition analysis. (**C**) HE staining. (**D**) Levels of *Sirt1* mRNA. (**E**) Levels of *Sirt1* AS lncRNA. (**F**) Levels of miR-34a. (**G/H**) Levels of *Sirt1*, CyclinB, CyclinD, CyclinE, MyoD, MyoG and MHC proteins. The results were presented as means ± SEM of at least 4 independent experiments. *P < 0.05, **P < 0.01. (**I**) Model for *Sirt1* AS lncRNA fully bound to *Sirt1* mRNA to regulate muscle formation by competing with miR34a. OE: *Sirt1* AS overexpression, Quad: quadriceps, Sol: soleus, Gast: gastrocnemius, Ing: inguinal fat.

To further confirm whether *Sirt1* AS lncRNA was implicated in muscle formation *in vivo*, mouse intraperitoneal injection of adenovirus-mediated *Sirt1* AS overexpression was performed. The results showed that mouse body weights decreased in *Sirt1* AS overexpression treatment than in the control at month 3, 4, 5 and 6 (Fig. 8A). Moreover, compared with the control, the lean mass was less in *Sirt1* AS overexpression treatment, whereas the fat mass did not change at month 6 (Fig. 8B). Muscle fiber size of quadriceps (Quad), soleus (Sol) and gastrocnemius (Gast) were apparently greater in *Sirt1* AS overexpression treatment than in the control, but fat cell size of inguinal fat (Ing) did not alter at month 6 (Fig. 8C). The levels of *Sirt1* mRNA and *Sirt1* AS lncRNA were upregulated in Quad, Sol and Gast in *Sirt1* AS overexpression treatment at month 6 (Fig. 8D,E). Interestingly, the levels of miR-34a did not change in Quad, Sol, Gast and Ing between the treatment and control (Fig. 8F). In addition, the protein levels of *Sirt1* and the proliferation genes including *CyclinB*, *CyclinD* and *CyclinE* increased, but protein levels of the differentiation gene including *MyoD*, *MyoG* and *MHC* decreased (Fig. 8G,H). Collectively, the above results suggested that *Sirt1* AS lncRNA was associated with myogenesis *in vivo*. Therefore, *Sirt1* AS lncRNA fully bound to *Sirt1* mRNA forming RNA duplex to regulate muscle formation by competing with miR34a (Fig. 8I).

## Discussion

The intrinsic nature and complex secondary structures of lncRNAs enable them to specifically interact with DNA, RNA and proteins. On the basement of relationship of *Sirt1* AS lncRNA, *Sirt1* mRNA and miR-34a sequences, we speculated that *Sirt1* AS lncRNA bound to *Sirt1* mRNA by competing with miR-34a to regulate the efficiency of *Sirt1* protein expression in myoblast proliferation and differentiation. Our results supported the above hypothesis. In this study, *Sirt1* AS lncRNA promoted myoblast proliferation by upregulating the expression of cell cyle genes including *CyclinB*, *CyclinD* and *CyclinE*, whereas it inhibited myoblast differentiation though repressing the expression of the myogenic factors *MyoD*, *MyoG* and *MHC*. The results were further confirmed by overexpression of *Sirt1* AS *in vivo*. Therefore, we uncovered the novel functional roles of *Sirt1* AS lncRNA in mediating muscle formation. As shown in Fig. 8I, functionally and mechanistically, *Sirt1* AS lncRNA fully bound to *Sirt1* mRNA to regulate muscle formation by competing with miR34a.

Although a large number of mammalian lncRNAs were pervasively identified by lncRNA-seq, only a minority were functionally explored. Our previous studies found that *PU.1* AS lncRNA was involved in adipogenesis^25^ and immunifaction^26^. In this study, our results clearly demonstrated that the expression of *Sirt1* AS lncRNA in myoblast proliferation was increased, which was similar with *Sirt1* mRNA, but opposite to miR-34a. It has been reported that miR-34a targeted *Sirt1* to inhibit *Sirt1* expression in embryonic stem cells^27^, neural stem cell^28^ and colon cancer cells^29^. Therefore, the expression patterns of *Sirt1* and miR-34a implied that miR-34a targets *Sirt1* in C2C12 cells. By luciferase reporter assay, we proved that both miR-34a and *Sirt1* AS lncRNA directly targeted *Sirt1* 3′ UTR. Interestingly, the levels of *Sirt1* and miR-34a were not strictly opposite, because more than 16 miRNAs regulate *Sirt1* expression and activity^30^, we thought that the other miRNAs as well were implicated in regulation of *Sirt1* expression in C2C12 proliferation. Moreover, miR-34a was not only targets *Sirt1* but also other genes^31^ to exert its biological functions. Surprisingly, *Sirt1* AS lncRNA resisted the roles of miR-34a in myoblast proliferation and differentiation, implying that interaction existed among *Sirt1* AS lncRNA, *Sirt1* mRNA and miR-34a.

In this study, we focused on *Sirt1* AS lncRNA and investigated relationship among *Sirt1* AS lncRNA, *Sirt1* mRNA and miRNA-34a in myogenesis. Based on our results, we found that *Sirt1* AS lncRNA preferably interacted with *Sirt1* mRNA forming RNA duplex to promote *Sirt1* translation by competing with miR-34a, inhibiting muscle formation. Up to now, the effects of *Sirt1* protein on myoblast proliferation and differentiation had been investigated very well^21^,^32^,^33^,^34^,^35^,^36^,^37^. In addition, our previous studies indicated the expression of *Sirt1* AS lncRNA in mouse various tissues including heart, kidney, liver, spleen, brain, muscle and white adipose tissue^24^. Therefore, we thought that the expression of *Sirt1* AS lncRNA was not localized to specific cell type.

Because lncRNAs are enriched in the nucleus or the cytosol, they can act at virtually every level of gene expression^38^,^39^. In this study, we found that *Sirt1* AS lncRNA was in both nucleus and cytoplasm, but its levels increased in myoblasts of *Sirt1* AS overexpression. Moreover, the RNA stability assay indicated *Sirt1* AS overexpression prolonged *Sirt1* mRNA half-life. The RPA assay further confirmed that *Sirt1* AS lncRNA bound to its mRNA forming mRNA/AS lncRNA compound in the myoblast cytoplasm. Taken together, we suggested that *Sirt1* AS lncRNA elevated translational efficiency of *Sirt1* mRNA by RNA duplex. However, whether nuclear *Sirt1* mRNA/AS lncRNA duplex affects sense RNA processing including capping, polyadenylation, nuclear localization and transport, will need further exploration.

Here, we need point out that interference of *Sirt1* AS lncRNA using RNAi will influence sense mRNA as well^40^, because *Sirt1* AS lncRNA is fully overlapping with *Sirt1* 3′ UTR by base complementary pairing principle. Additionally, AS transcripts including *Sirt1* AS lncRNA are generally low in abundance and are, on average, more than 10-fold lower in abundance than sense expression^40^,^41^,^42^. It was why we overexpressed *Sirt1* AS lncRNA to explore its myogenic function, but not interfered in this study.

Because the target site of miR-34a located in *Sirt1* mRNA and AS lncRNA overlapping region, the function of miR-34a was studied as well. As expected, miR-34a overexpression decreased the levels of *Sirt1* expression and suppressed C2C12 cell proliferation but promoted the cell differentiation. Whether *Sirt1* AS lncRNA interacted with miR-34a regulates *Sirt1* expression was still unknown. Recent studies showed that lncRNAs involved in miRNAs processing regulation^16^,^17^. In addition, lncRNA also influenced miRNAs function by directly “sponging”^11^,^15^. Interestingly, we here found that *Sirt1* AS lncRNA bound to *Sirt1* 3′ UTR by competing with miR-34a to attenuate the functional role of miR-34a. Therefore, we thought that AS lncRNA regulated cognate gene mRNA through competing with miRNA may be an extensive novel mechanism.

AS lncRNA is an important class of lncRNAs, which transcripts from antisense strand^40^. AS lncRNAs which were partially or fully complementary to sense mRNA in the nucleus or/and cytoplasm, decided their specific functional mechanisms^1^. For example, apolipoprotein A1 (*APOA1*) AS lncRNA can regulate the histone methylation patterns of sense *APO* gene cluster through guiding histone-modifying enzymes lysine (K)-specific demethylase 1 (LSD1) or suppressor of zeste 12 homolog (SUZ12) to *APO* gene cluster^42^. Ubiquitin carboxy-terminal hydrolase L1 (*Uchl1*) AS lncRNA regulated Uchl1 translation through an embedded inverted short interspersed nuclear element B2 (SINEB2) element which activated polysomes^43^. AS lncRNA and mRNA formed double strand duplex to recruit Staufen 1 (STAU1) protein to mRNAs and mediate their degradation^44^. In our study, *Sirt1* AS overexpression increased *Sirt1* mRNA stability, implying that *Sirt1* AS lncRNA regulated *Sirt1* expression at translational level. Moreover, we found that *Sirt1* AS lncRNA bound to *Sirt1* 3′ UTR though competing with miR-34a to resist downregulation of *Sirt1* level resulting from miR-34a.

In the cytoplasm, lncRNAs affect translational output in different ways. They can control gene expression by reducing or stimulating mRNA decay^44^,^45^. A particular class of cytoplasmic lncRNAs, the competing endogenous RNAs (ceRNA), regulates both the translation and the degradation rates of mRNAs by acting as molecular “sponges” for miRNAs, thus modulating the repressive activity of miRNA on their mRNA targets^46^,^47^,^48^,^49^,^50^. On the basement of our above findings, we here defined a novel regulatory mechanism of AS lncRNA, which is that AS lncRNA can also make mRNA stability and compete with miRNA to influence translational output to regulate muscle formation.

Intraperitoneal injection of adenoviral vectors was validated as an efficient gene manipulation tool for overexpressing recombinant proteins *in vivo*^51^,^52^. Of course, gene editing (CRISP/Cas9) and transgenic technologies may be also the good methods. Moreover, our lab first identified *Sirt1* AS lncRNA and investigated its stability and expression. Fortunately, we also found that *Sirt1* AS lncRNA was elevated more than 40-fold by resveratrol (an agonist of *Sirt1*^53^,^54^). Based on our findings, we think that *Sirt1* AS lncRNA may be a biomarker which indicates muscle formation.

Collectively, our findings unravel a novel molecular mechanism on muscle formation by *Sirt1* AS lncRNA. Mechanically, full complementary binding of *Sirt1* AS lncRNA to *Sirt1* mRNA forming RNA duplex in the cytoplasm facilitated *Sirt1* translation output by competing with miR-34a, inhibiting myogenesis via promoting myoblast proliferation and inhibiting differentiation. The elucidation of mechanism on *Sirt1* protein output by *Sirt1* AS lncRNA will provide insight into novel pathways of regulatory myogenesis.

## Methods

### Cell culture and transfection

C2C12 cell line was purchased from ATCC (Rockville, MD, USA) and cultured in Dulbecco’s modified Eagle’s medium (DMEM: Gibco, Carlsbad, CA) supplemented with 10% fetal bovine serum (FBS: Hyclone Laboratories, Logan, UT) at 37 °C and 5% CO_2_. After cells reached confluence, they were induced to myogenic differentiation by replacing 10% FBS with 2% horse serum (Gibco, Carlsbad, CA). For cell proliferation detection, cells were transfected with *Sirt1* AS lncRNA, miR-34a, *Sirt1* AS lncRNA plus miR-34a and pCDNA3.1 empty plasmids using Lipofectamine 2000 (Life Technologies, Shanghai, China) according to the manufacturer’s instructions when the cells reached 50–60% confluence after seeding for 12 hours. Twenty-four hours post-transfection, the cells were harvested and preserved at −80 °C for further proliferation assay. For cell differentiation analysis, cells were seeded in six-well plates with culture medium and transfected with above plasmids on the first day of myogenic differentiation. Forty-eight hours post-transfection, they were harvested and preserved at −80 °C for further myogenic differentiation detection.

### Real-time qPCR

Cell total RNA was extracted according to the standard method with a TRIzol reagent (Takara, Kyoto, Japan). Reverse transcription was performed to synthesize cDNA by using the RT Kit (Takara, Kyoto, Japan). The primers used for real-time qPCR were designed and synthesized (Table S1). Real time qRT-PCR was carried out in IQ5 real-time PCR detection system (Bio-Rad, Hercules, CA) using SYBR-Green (Vazyme). The mRNA data was quantified using the comparative threshold cycle (ΔΔCT) methods.

### Strand-specific RT-PCR

Strand-specific RT-PCR was performed as our previous publications^18^,^26^. Briefly, before reverse transcription reactions, 1μg cells total RNA was performed to wipe out DNA contamination by gDNA wiper Mix (Vazyme) at 42 °C for 2 min. Reverse transcription reactions were carried out using RT kit (Vazyme). The strand-specific primers, reverse primer of *Sirt1* AS lncRNA or miR-34a RT primer as the reverse transcription primers. The RT was at 50 °C for 30 min, 85 °C for 5 s in 10 μl volumes.

### Cytoplasmic and nuclear lncRNA

RNAs were extracted from C2C12 cells according to merchant guide using Nuclear or Cytoplasmic RNA Purification Kit (Fisher BioReagents) as previously described^55^. Briefly, cell pellet was resuspended in buffers of RNA Purification Kit and followed by centrifugation at 4 °C twice. The supernatant was saved as cytoplasmic fraction and the pellet was used as nuclear fraction. RNAs were extracted from both fractions using Trizol. The relative levels of *Sirt1* AS lncRNA and mRNA were analyzed by real-time qPCR.

### Western blotting

The cellular protein was extracted in the RIPA lysis buffer (Appligen, Beijing, China) with 1 mM PMSF. Peterson’s method was used to quantify the protein contents. Western blotting were performed as our previously described^23^. Following primary antibodies were used: *Sirt1* (1:500; Santa Cruz Biotechnology, Dallas, TX), CyclinD (1:500; Boster, Wuhan, China), CyclinB (1:500; Santa Cruz Biotechnology, Dallas, TX), CyclinE (1:1000; Santa Cruz Biotechnology, Dallas, TX), MyoD (1:1000; Santa Cruz Biotechnology, Dallas, TX), MyoG (1:1000; Santa Cruz Biotechnology, Dallas, TX), MHC (1:2000; Sigma, St. Louis, MO) and GAPDH (1:1000; Santa Cruz Biotechnology, Dallas, TX). The secondary antibodies were anti-rabbit, anti-goat and anti-mouse antibodies (Santa Cruz Biotechnology, Dallas, TX). The targeted proteins were detected using the Gel Doc XR System and analysis software Quantity One (Bio-Rad, Hercules, CA) as per the instructions of the manufacturer.

### Flow cytometric analysis

Flow cytometric analysis was carried out as our previous publication^56^. Twenty-four hours post-transfection, the cells were washed 3 times with cold PBS and fixed in 70% (v/v) cold ethanol overnight. Then centrifugation and the supernatants were discarded, the cells were washed with PBS again and resuspended in 500μl propidium iodide (50 μg/ml; DOJINDO, Shanghai, China) solution with 100 μg/ml RNase A and 0.2% (v/v) TritonX-100 at 4 °C for 30 min. The cells were detected with a flow cytometry instrument (Becton Dickinson, FACSCa-libur, Franklin, GA). Cell cycle phases were assigned to G0/G1, S, and G2/M according to the amount of DNA. The proportion of cells in each cell cycle phase was statistically analyzed.

### CCK-8 proliferation detection

C2C12 cells were seeded in 96-well plates at 4 × 10^3^ cells per well with 100 μl DMEM. The Cells were transfected with *Sirt1* AS lncRNA, miR-34a, *Sirt1* AS lncRNA plus miR-34a and pCDNA3.1 empty plasmids. Twenty-four hours post-transfection, cell proliferation index was detected using CCK-8 kit (Beyotime, Shanghai, China) according to manufacturer’s instructions.

### EDU proliferation analysis

Twenty-four hours post-transfection, C2C12 cells were treated with 10 μM EdU (Ribobio) and incubated for 3 h. EDU staining were processed according to the manufacturer’s protocol. Cell nucleus was stained with 5 μg/ml DAPI (Roche, Penzberg, Germany) for 10 min. The cells were visualized by a fluorescence microscope (Nikon, Tokyo, Japan). The ratio of positive cells (EdU-staining cells/the total of cells) was calculated.

### Immunocytochemistry

Immunocytochemistry were performed according to the recently published method^55^. Briefly, cultured cells were fixed with 4% paraformaldehyde for 10 min at 4 °C, washed 3 times with PBS, and permeabilized using 0.3% TritonX-100 in TBS. Following blocking with 2% goat serum in TBS for 1 h at room temperature, the cells were incubated with primary antibodies MHC (1:200) or MyoG (1:200) at 4 °C overnight with gentle shaking, washed, and then incubated with secondary antibodies (AlexaFluor-488 conjugated mouse IgG1 and AlexaFluor-568 conjugated mouse IgG2b (1:1000, Invitrogen)) for 1 h at room temperature. Cells were then washed 3 times with TBST, stained with DAPI, and photographed using a NIKON TE2000-U fluorescent microscope (Nikon, Tokyo, Japan).

### Luciferase reporter assay

The partial 3′ UTR of mouse *Sirt1* mRNA was amplified by PCR and inserted into the psiCHECK™-2 Vector (Promega, Madison, USA). The primers used to construct plasmids for luciferase reporter assay were shown in Table S2. *Sirt1* AS lncRNA and miR-34a expression plasmids were co-transfected with *Sirt1* 3′ UTR psiCHECK™-2 vector into 293T cells by Transfection Reagent (Roche, Penzberg, Germany), respectively. Forty-eight hours post-transfection, the cells were harvested, incubated with cell lysis buffer, and the luciferase activity was then measured using ELIASA (PerkinElmer, Waltham, MA) according to the manufacturer’s instructions.

### RNA stability assay

To detect the stability of *Sirt1* mRNA, C2C12 cells were treated with 2μg/ml Actinomycin D (Sigma-Aldrich, Louis, MO) which could suppress transcription. The cells were harvested at 0, 1, 2, 6 and 10 h post treatment, and total cellular RNA was extracted to detect the residual mRNAs by real time qPCR. GAPDH mRNA was acted as internal control, because was stable within 32 h.

### Ribonuclease protection assay

To detect the sense-antisense RNA duplex, ribonuclease protection assay (RPA) and RT-PCR were performed on the total RNA from C2C12 cells. This step was conducted according to the previous studies^57^,^58^ with a few changes. Sequence of oligonucleotides used for RPA was listed in Table S3. Cytoplasmic RNA was orderly digested by DNaseI (Fermentas) and RPA-grade RNaseA (Applied Biosystems) to remove all the genomic DNA contamination and single-strand RNAs. Then the residues, endogenous double-strand RNAs (dsRNA) were applied in the RT reaction catalyzed by the reverse transcriptase Superscript III (Invitrogen). The reaction system was incubated at 55 °C for 60 min and terminated at 75 °C for 10 min. Finally, the double-strand cDNA was amplified in 25 μl PCR reaction system. After 35-cycle amplification, the products were checked by electrophoresis on 1.5 % agarose gel with ethidium bromide staining.

### Animal studies

C57B/L6 mice were housed in the animal facilities of Northwest A&F University under conventional conditions with constant temperature and humidity and fed a standard diet. All mice experiments were carried out in accordance with the protocol approved by the Animal Ethics Committee of Northwest A&F University and the experimental protocol was performed in accordance with applicable guidelines and regulations. Intraperitoneal injection of adenoviral vectors was validated as an efficient gene manipulation tool for overexpressing recombinant proteins *in vivo*^51^. For treatment with adenovirus-mediated *Sirt1* AS *in vivo* according to our previous publication^52^, 2-month-old mice were injected with 500 μl adenovirus (Ad-*Sirt1* AS or Ad-EGFP) into the cavitas abdominalis every 10 days until 180 days of age. Whole body composition analysis was carried out per month using EchoMRI-100 (Houston, TX, USA). Mice were sacrificed and muscle and fat tissues were harvested on day 180, and total RNAs and proteins were extracted for real-time RT-PCR and western blotting analyses. Mice (4~6) were used in each group.

### Frozen section and HE staining

For Frozen section and HE staining of mouse Quad, Sol, Gast, Ing sections were collected on day 180 for *Sirt1* AS lncRNA overexpression. Frozen section and HE staining were performed according to the recently published method^59^. The sections were observed and taken through pictures with microscope (Olympus, New York, NY).

### Statistical analysis

All experimental data were obtained through at least four independent experiments. Values were showed as means ± SEM. Statistical analysis was performed in GraphPad Prism version 6 (GraphPad software, La Jolla). Student’s *t* test is used for individual comparisons. Multiple comparisons are assessed by one-way ANOVA followed by Dunnett’s tests. Difference between groups was considered statistically significant if *P* < 0.05.

## Additional Information

**How to cite this article**: Wang, G.-q. *et al.* *Sirt1* AS lncRNA interacts with its mRNA to inhibit muscle formation by attenuating function of miR-34a. *Sci. Rep.* **6**, 21865; doi: 10.1038/srep21865 (2016).

## Supplementary Material

Supplementary Information
