# Supplementary material for: Sirt1 AS lncRNA interacts with its mRNA to inhibit muscle formation by attenuating function of miR-34a
Source: Sci Rep. 2016 Feb 23;6:21865. doi: 10.1038/srep21865 (PMC4763196; doi:10.1038/srep21865)
Supplement: Supplementary Information [file srep21865-s1.pdf]

Sirt1 AS lncRNA interacts with its mRNA to inhibit muscle formation by attenuating function of miR-34a

Guo-qiang Wang [#], Yu Wang [#], Yan Xiong [#], Xiao-Chang Chen, Mei-ling Ma, Rui Cai, Yun Gao, Yun-mei Sun, Gong-She Yang & Wei-Jun Pang *

Laboratory of Animal Fat Deposition & Muscle Development, College of Animal Science and Technology, Northwest A&F University, Yangling Shaanxi 712100, China

Running Title: _Sirt1_ AS lncRNA inhibits muscle formation

[#] These authors contributed equally to this work

* **Correspondence:** Wei-Jun Pang Ph.D.

Address: No. 22 Xinong Road, Yangling, Shaanxi Province 712100, China

Tel: 86-29-87091017

Fax: 86-29-87092430

E-mail: pwj1226@nwsuaf.edu.cn

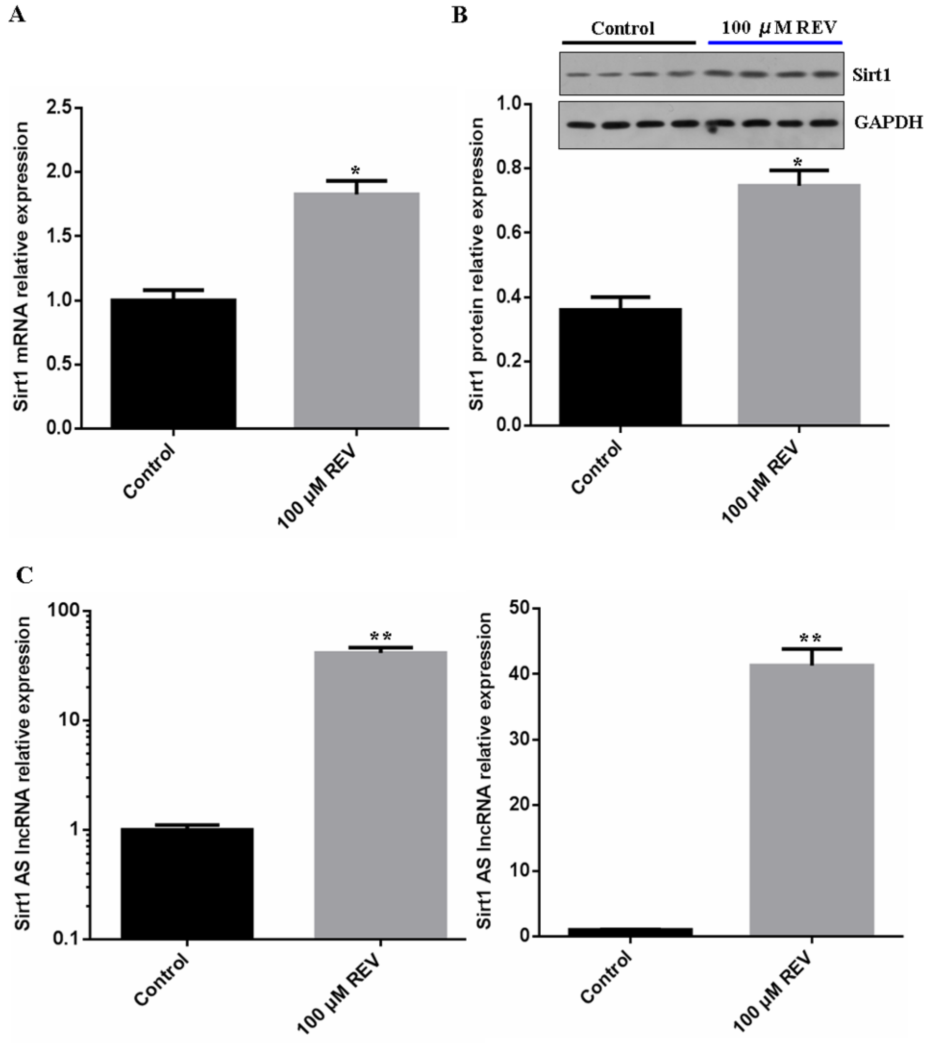

Figure S1. Resveratrol upregulates *Sirt1*AS lncRNA in myoblasts. C2C12 myoblasts were induced with 100μM resveratrol (REV; Sigma) according to the manufacturer's instructions when the cells reached 50-60% confluence after seeding in 6-well culture plates for 12 hours. Forty-eight hours post-induction, the cells were harvested and total RNA and protein were extracted for further strand-specific RT-PCR and western blot analysis. (**A**) Expression of *Sirt1* mRNA. (**B**) Expression of Sirt1 protein. (**C**) Expression of *Sirt1*AS lncRNA. The different histogram using log (left) and non-log (right) analysis in GraphPad Prism version 6. The data were presented as means ± SEM of 4 to 6 independent experiments. *P < 0.05 and **P < 0.01.

Table S1. Nucleotide sequences of the primers used for real-time quantitative PCR.

Genes	Direction	Primer sequence
Sirt1 AS	forward	5'-AATCCAGTCATTAAACGGTCTACAA-3'
	reverse	5'-TAGGACCATTACTGCCAGAGG A-3'
miR-34a	forward	5'-CACGCATGGCAGTGTCTTAGC-3'
	reverse	5'-CAGTGCAGGGTCCGAGGTA-3'
CyclinB	forward	5'-AACTTCAGCCTGGGTCG-3'
	reverse	5'-CAGGGAGTCTTCACTGTAGGA-3'
CyclinD	forward	5'-TAGGCCCTCAGCCTCACTC-3'
	reverse	5'-CCACCCCTGGGATAAAGCAC-3'
CyclinE	forward	5'-CAGAGCAGCGAGCAGGAGC-3'
	reverse	5'-GCAGCTGCTTCCACACCACT-3'
MyoD	forward	5'-CGGCTCTCTCTGCTCCTTTG-3'
	reverse	5’-GTCGAAACACGGGTCATCA-3’
MyoG	forward	5'-GACCCTACAGACGCCCACAA-3'
	reverse	5'-CCGTGATGCTGTCCACGAT-3'
MHC	forward	5'-CGCAAGAATGTTCTCAGGCT-3'
	reverse	5'-GCCAGGTTGACATTGGATTG-3'
GAPDH	forward	5'-TGCTGAGTATGTCGTGGAGTCT-3'
	reverse	5'-ATGCATTGCTGACAATCTTGAG-3'

Table S2. The primers used to construct plasmids for luciferase reporter assay.

Genes	Direction	Primer sequence
Sirt1 AS	forward	5'-CGGGGTACCTATGCTATGAACAATGGAAG-3'
	reverse	5'-CCGCTCGAGTTGCCTGTTGAGGATTTGGT-3'
miR-34a	forward	5'-CGGGGTACCCTGCTGTACCCTGCTGCTT-3'
	reverse	5'-CCCAAGCTTTGGGCGTTGCTGACCTCT-3'
Sirt1 3′ UTR	forward	5'-CCGCTCGAG TTGCCTGTTGAGGATTTGGT-3'
	reverse	5'-ATTTGCGGCCGCTATGCTATGAACAATGGAAG-3'

Table S3. Sequence of oligonucleotides used for RPA.

Genes	Direction	Primer sequence	Product length, bp
Sirt1	P1	5'-CTAGGTGGTGAATATGCCAAAC-3'	130
	P2	5'-TCCGAAATATGAAGAGGTGTTG-3'	
	P3	5'-GGTAGAGCCTGCATAGATCTT-3'	178
	P4	5'-ATTTATCTTATGACTTTGGGACTT-3'	
β-actin	forward	5'-TGCTGTCCCTGTATGCCTCTG-3'	223
	reverse	5'-TTGATGTCACGCACGATTTCC-3'	
